# Pretreatment With Risperidone Ameliorates Systemic LPS-Induced Oxidative Stress in the Cortex and Hippocampus

**DOI:** 10.3389/fnins.2018.00384

**Published:** 2018-06-08

**Authors:** Md. Mamun Al-Amin, Md. Faiyad Rahman Choudhury, Al Saad Chowdhury, Tahsinur Rahman Chowdhury, Preeti Jain, Mohsin Kazi, Musaed Alkholief, Sultan M. Alshehri, Hasan Mahmud Reza

**Affiliations:** ^1^Department of Pharmaceutical Sciences, North South University, Dhaka, Bangladesh; ^2^Department of Pharmaceutics, College of Pharmacy, King Saud University, Riyadh, Saudi Arabia

**Keywords:** brain, psychiatric disease, oxidative stress, risperidone, LPS

## Abstract

Risperidone (RIS), an atypical antipsychotic has been found to show anti-inflammatory effect against lipopolysaccharide (LPS)-induced inflammation. *In vitro* study has revealed that RIS inhibits the LPS-induced reactive oxygen species (ROS) formation. We investigated the antioxidant effects of RIS on LPS-induced oxidative stress markers in *Swiss albino* mice. Ten weeks old male *Swiss albino* mice (30 ± 2 g) were pretreated with either distilled water (control) or RIS (3 mg/kg) for 7 days. On day 8, animals were challenged with a single dose of LPS (0.8 mg/kg) while control animals received distilled water only. The animals were sacrificed after 24 h of LPS administration and tissue samples were collected. RIS administration significantly (*p* < 0.05) reduced the LPS-induced elevated levels of lipid peroxidation product malondialdehyde (MDA), advanced protein oxidation products, and nitric oxide (NO) in the cortex. Catalase (CAT) and superoxide dismutase (SOD) levels were also diminished while the level of glutathione (GSH) was enhanced. Hippocampus data showed that RIS significantly (*p* < 0.05) reduced the LPS-induced increased levels of MDA and NO, and SOD activity. Our results suggest that LPS-induced neuronal oxidative damage can be alleviated by the pretreatment with RIS and the effect is shown presumably by scavenging of the ROS by risperidone as an antioxidant.

## Introduction

The brain is a specialized restricted region separated by blood–brain barrier (BBB). Endothelial cells, astrocytes, and microglial cells are the vital elements that conserve the features of BBB. Endothelial cells create a tight junction and electrical resistance of BBB, thus selectively permitting external substances to penetrate through. On the other hand, astrocytes and microglial cells provide a distinct immune mechanism to the brain. However, under inflammatory state, BBB becomes leaky and allows the access of undesirable components, such as immune cells, inflammatory molecules, and albumin to the brain. Entrance of pro-inflammatory cytokines; tumor necrosis factor-alpha (TNF-α), interleukin-6 (IL-6) has been shown to be associated with the progression of Parkinson’s disease (Smith et al., 2012), Alzheimer’s disease (Minagar et al., 2002), and schizophrenia (O’Brien et al., 2008; Al-Amin and Reza, 2014). Inflammatory cytokines (IL-1 or IL-6, TNF-α); nitric oxide (NO); and reactive oxygen species (ROS) may also mediate neurodegeneration (Gebicke-Haerter, 2001; Vallieres et al., 2002). Under inflammatory condition, microglia becomes activated resulting in the release of cytotoxic mediators; such as NO, TNF-α, IL-1β, and ROS. Overproduction of these mediators has been found to be toxic to the neurons which leads to neuronal death (Cui et al., 2012).

Inflammatory agents such as LPS (lipopolysaccharide) has been used to simulate a range of brain diseases; depression (Medeiros et al., 2015), schizophrenia (Zhu et al., 2014a), autism (Kirsten et al., 2015), Parkinson’s disease (Sharma and Nehru, 2015), Alzheimer’s disease (El-Sayed and Bayan, 2015) in rodents. LPS is a major component of the cell wall of gram negative bacteria and a potent activator of the inflammatory response, particularly of microglia. LPS at a dose of 1 g/ml (intraperitoneal injection) enhances the permeability of the BBB and increases the production of ROS in the brain (Zhao et al., 2014; Yu et al., 2015). While, most of the studies have discussed the inflammatory mechanism of LPS, several studies have explained LPS-induced oxidative damage in the brain (Noworyta-Sokolowska et al., 2013). For example, ROS formation is accelerated by LPS, which causes a significant alteration in NO, malondialdehyde (MDA), GSH, superoxide dismutase (SOD), and CAT levels in brain disease (Sharma and Nehru, 2015). Previous studies have shown that an increased level of pro-inflammatory cytokines (e.g., TNF-α, INF-γ, and IL-6) can be the basis of formation of ROS, MDA, AOPP, CAT, SOD, and GSH (Zhang et al., 2003; Rukmini et al., 2004; Yao et al., 2006; Dean et al., 2009).

Peripheral systemic administration of LPS has shown to produce neuro-inflammation and oxidative stress in the brain (Ho et al., 2015). Single-dose of LPS is adequate to stimulate the production of inflammatory cytokines; IL-1β, IL-6, and TNF-α in the prefrontal cortex and hippocampus (Sulakhiya et al., 2014; Ho et al., 2015; Li et al., 2015). Previous research has indicated that a single dose of systemic LPS could enhance the production of MDA – a lipid peroxidation marker, nitrite- a reactive nitrogen species marker, while reduce GSH level in the rat (Shukla et al., 2008). On the other hand, RIS, a second generation atypical antipsychotic, which is prescribed for the treatment of schizophrenia, schizoaffective disorders, bipolar disorders, and behavioral irritability in autistic patients, was used to improve the situation given that the anti-inflammatory activity of RIS is mediated through the reduction of TNF-α, INF-γ, and IL-6 (Al-Amin et al., 2013; MacDowell et al., 2013; Ajami et al., 2014), and prevention of microglial activation (Zhu et al., 2014b). The antioxidant effect of RIS has been reported previously. *In vitro* human U937 cell culture study by Chen et al. (2013) revealed that RIS inhibits LPS-induced ROS formation. A recent study on first episode drug naïve schizophrenic patient demonstrates that 11 weeks-long treatment with RIS lowers lipid peroxidation (Noto et al., 2015). Moreover, RIS treatment reduces elevated SOD level in schizophrenic patient (Zhang et al., 2012). In animal model of schizophrenia, RIS has been found to enhance the level of GSH, while reduce SOD and MDA (Stojković et al., 2012) levels. Neuroprotective properties exhibited by RIS could be attributed to microglial activation or antioxidant maintenance mechanisms in the cerebral cortex and hippocampus (Yan et al., 2014). The aim of this study was to investigate the effect of risperidone (RIS) on the LPS-induced oxidative stress in the prefrontal cortex and hippocampus.

## Materials and Methods

### Animals

Male *Swiss albino* (10 weeks old weighing 30–32 g) mice (*n* = 24) were obtained and 6 mice per cage (Tecniplast, Italy) were housed in humidity controlled environment at a temperature of 25 ± 1°C on a 12 h light/day cycle, with liberal access to food and water. Prior to the testing, the mice were allowed to get habituated to the testing rooms for 1 week. The mice were divided into four groups; Ctrl (control) (*n* = 6), LPS (lipopolysaccharide, I.P.) (*n* = 6), RIS (risperidone) (*n* = 6), and RIS+LPS (risperidone + LPS) (*n* = 6). RIS was administered at a dose of 3 mg/kg once daily, for 7 days. LPS (*Escherichia coli*, Sigma Aldrich), was administered only once on day 8 at a concentration of 0.8 mg/kg with the water for injection (WFI). All experimental procedures were approved by the institutional ethical committee, North South University (NSU/PHA/2014/133-046), Dhaka, Bangladesh. Animals were handled in accordance with the international principles guiding the usage and handling of experimental animals (United States National Institute for Health Publication, 1985).

### Preparation of RIS and LPS

Risperidone powder (99.8%) was obtained as a gift from General Pharmaceuticals, Ltd., Dhaka, Bangladesh. RIS was dissolved in distilled water and administered orally at a dose of 3 mg/kg (O’Sullivan et al., 2014). LPS (10 μg/μl) (Zhu et al., 2014b) was dissolved in WFI and a single dose was administered via IP route. The concentration of the administered risperidone dose was 900 μg/ml. The control animals received 100 μl of distilled water orally. LPS group received LPS at a dose of 0.8 mg/kg body weight. The working concentration of LPS was 240 μg/mL. LPS + Risperidone group received LPS (0.8 mg/kg) and Risperidone (3 mg/kg). Risperidone group received risperidone at a dose of 3 mg/kg.

### Tissue Collection

Mice were euthanized using 200 μl of ketamine (50 mg/ml, ACI Pharmaceuticals, Ltd., Bangladesh). The animals were sacrificed by decapitation. The entire brain was rapidly removed cautiously and kept in a Petri dish on ice. Then cortex, hippocampus regions were dissected from the brain. Liver tissue was also collected. Homogenate of various brain regions, 10% (w/v) were prepared in phosphate buffer saline (PBS) (10 mM, pH 7.0) using Ultra-Turrax T25 (United States) homogenizer. Homogenized tissue samples were sonicated at 5 s cycle for 30 s using an ultrasonic processor and centrifuged at 10,000 rpm (RCF 11200) for 10 min. The supernatant was diluted with 0.1x PBS buffer and preserved in -20°C. The clear supernatants were collected for the biochemical analysis.

### Biochemical Test

The following biochemical tests were conducted in triplicate.

#### Determination of the Level of MDA

Lipid peroxidation was evaluated colorimetrically as described previously (Niehaus and Samuelsson, 1968). Briefly, 0.1 ml of tissue homogenate (Tris-HCl buffer, pH 7.5) was treated with 2 ml of (1:1:1 ratio) TBA-TCA-HCl reagent (2-thiobarbituric acid 0.37%, 0.25 N HCl and 15% TCA) and placed in water bath (70°C) for 15 min and cooled. The absorbance of clear supernatant was measured against reference blank at a wavelength of 535 nm. The level of MDA was measured by using standard curve and expressed as nmol/mg of tissue.

#### Determination of the Level of APOP

Determination of advanced protein oxidation products (APOPs) was carried out spectrophotometrically as described by Witko-Sarsat et al., 1996). Concisely, 50 μl of plasma, which was diluted 1:2 with PBS and chloramine T (0–100 μmol/l) were used for the preparation of calibration curve. PBS was used as blank. One hundred μl of 1.16 M potassium iodide and 50 μl of acetic acid were added to each well and absorbance was measured immediately at a wavelength of 340 nm. Concentration of APOP was expressed as μmol/mg of tissue.

#### Determination of the Level of NO

Nitric oxide was assayed according to the method described by Tracey et al. (1995). In this assay, Griess-Illosvoy reagent was modified by using naphthyl ethylene diaminedihydrochloride (0.1% w/v) instead of 1-napthylamine (5%). The reaction mixture (3 ml) containing brain homogenates (1.5 ml) and PBS (1.5 ml) was incubated at 25°C for 15 min. Rest of the process was followed as described previously (Al-Amin et al., 2015). A pink colored chromophore was formed in diffused light. The absorbance was measured at a wavelength of 540 nm against the corresponding blank. NO level was determined by using standard curve and expressed as nmol/mg of tissue.

#### Determination of the Activity of CAT

The activity of catalase enzyme was assayed spectrometrically at a wavelength of 240 nm (Sinha, 1972). The reaction mixture (1.5 ml) contained 1.0 ml of 0.01 M phosphate buffer (pH 7.0), 0.1 ml of tissue homogenate (supernatant) and 0.4 ml of 2 M H_2_O_2_. The reaction was stopped by the addition of 2.0 ml of dichromate-acetic acid reagent (5% potassium dichromate and glacial acetic acid were mixed in 1:3 ratio). The activity of catalase was expressed as the percent change in absorption between initial and subsequent at 1 min interval.

#### Determination of the Activity of SOD

The activity of SOD was assayed by a modified procedure by Ma et al. (2010). Briefly, 300 μL of reaction mixture contained 50 mM sodium phosphate (pH 7.8), 13 mM methionine, 75 mM nitrobluetetrazolium (NBT), 2 mM riboflavin, 100 mM EDTA, and 20 μl of plasma. The change in absorbance of the sample was then recorded at a wavelength of 560 nm after the production of blue formazan. The activity of SOD was determined as changes of absorption between initial and subsequent at 30 s interval divided by initial absorption. Results were expressed as percentage activity of SOD enzyme.

#### Determination of the Level of GSH

Glutathione in the brain was evaluated based on the method described by Ellman (1959). Briefly, 1 ml of plasma was added with 2.7 ml of phosphate buffer (0.1 M, pH 8) and 0.2 ml of 5, 5-dithio-bis (2-nitrobenzoic acid). The color developed was determined immediately at 412 nm. Results of glutathione assay was expressed as μmol/mg of protein.

### Data Analysis

All the biochemical tests were carried out in triplicate and data were represented as mean ± SEM (standard error of mean). One-way ANOVA was conducted followed by suitable *post hoc* test to analyze the main effect of treatment on the dependent variables among four treatment groups. Oxidative stress markers (MDA, APOP, NO, GSH, CAT, and SOD) were considered as dependent variables. All analyses were carried out in SPSS 16, and graphs were prepared using GraphPad prism (version 6.0, GraphPad Software, Inc.). The difference was considered significant when *p*-value was at least less than 0.05.

## Results

### Effect of Risperidone on Lipid Peroxidation (MDA)

**FIGURE 1:**
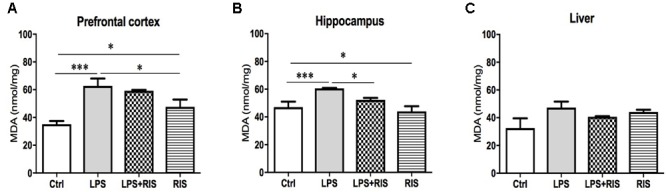
Effect of various treatments on the level of lipid peroxidation (MDA). Groups are Ctrl (control), LPS (lipopolysaccharide), LPS+RIS (lipopolysaccharide + risperidone), and RIS (risperidone). The level of MDA was assayed from the prefrontal cortex **(A)**, hippocampus **(B)**, and liver **(C)** tissues. Values are represented as mean ± SEM. *Post hoc* multiple comparison test namely, “Newman–Keuls” test was used to compare between groups. *n* = 6 per group. ^∗^*p* < 0.05, ^∗∗^*p* < 0.01, ^∗∗∗^*p* < 0.001.

There was a significant main effect of risperidone treatment on MDA level [*F*_(3,20)_ = 10.04, *p* < 0.001] in the prefrontal cortex (**Figure 1A**). *Post hoc* multiple comparison test indicated an increased level of MDA in the LPS group (*M* = 62.67, *SD* = 13.15) than the control (*M* = 35.11, *SD* = 5.72), LPS+RIS (*M* = 59.17, *SD* = 1.64) and RIS (*M* = 47.67, *SD* = 12.78) groups.

There was a noticeable main effect of treatment on MDA level [*F*_(3,20)_ = 6.70, *p* = 0.002] in the hippocampus (**Figure 1B**) also. *Post hoc* analysis exhibited an increased level of MDA in the LPS (*M* = 60.67, *SD* = 0.73) than the control (*M* = 47.00, *SD* = 9.83) group. Interestingly, LPS+RIS (*M* = 52.33, *SD* = 3.28) group had a lower level of MDA than LPS (*M* = 60.67, *SD* = 0.73) group. However, there was no significant effect of treatment on MDA [*F*_(3,18)_ = 2.75, *p* = 0.07] level in the liver (**Figure 1C**).

### Effect of Risperidone on Advanced Protein Oxidation Product (APOP)

**FIGURE 2:**
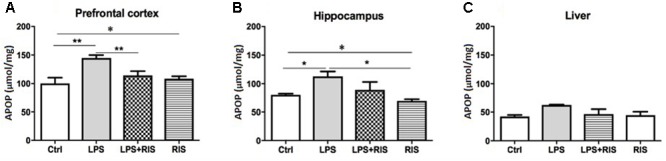
Effect of various treatments on the level of advanced protein oxidation products (APOPs). Groups are Ctrl (control), LPS (lipopolysaccharide), LPS+RIS (lipopolysaccharide + risperidone), and RIS (risperidone). The level of AOPP was determined from the prefrontal cortex **(A)**, hippocampus **(B)**, and liver **(C)** tissues. Values are represented as mean ± SEM. *Post hoc* multiple comparison test namely, “Newman–Keuls” test was used to compare between groups. *n* = 6 per group. ^∗^*p* < 0.05, ^∗∗^*p* < 0.01, ^∗∗∗^*p* < 0.001.

There was a significant main effect of treatment on APOP [*F*_(3,20)_ = 7.66, *p* < 0.001] in the prefrontal cortex (**Figure 2A**). *Post hoc* analysis showed an enhanced level of AOPP in the LPS (*M* = 144.7, *SD* = 12.86) than the control (*M* = 100.2, *SD* = 23.71) group. LPS+RIS (*M* = 113.8, *SD* = 18.56), group showed a lower level of MDA than LPS (*M* = 144.7, *SD* = 12.84) group indicating effectiveness of risperidone treatment.

The noticeable main effect of treatment on AOPP [*F*_(3,20)_ = 4.63, *p* < 0.01] in the hippocampus is shown in **Figure 2B**. *Post hoc* test displayed an elevated level of AOPP in the LPS (*M* = 112.3, *SD* = 21.44) than control (*M* = 80.33, *SD* = 4.92), LPS+RIS (*M* = 89.17, *SD* = 34.25), and RIS (*M* = 69.50, *SD* = 7.66) groups. No significant main effect of treatment on AOPP in the liver [*F*_(3,18)_ = 2.67, *p* = 0.07] was observed (**Figure 2C**).

### Effect of Risperidone on Nitric Oxide (NO)

**FIGURE 3:**
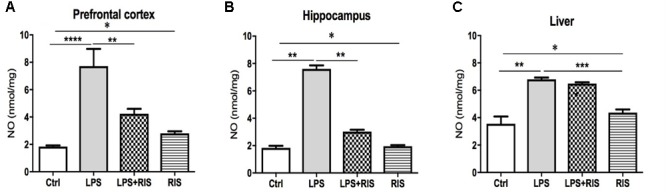
Effect of various treatments on the level of nitric oxide (NO). Groups are Ctrl (control), LPS (lipopolysaccharide), LPS+RIS (lipopolysaccharide + risperidone), and RIS (risperidone). NO was measured from the prefrontal cortex **(A)**, hippocampus **(B)**, and liver **(C)** tissues. Values are represented as mean ± SEM. *Post hoc* multiple comparison test namely, “Newman–Keuls” test was used to compare between groups. *n* = 6 per group. ^∗^*p* < 0.05, ^∗∗^*p* < 0.01, ^∗∗∗^*p* < 0.001, ^∗∗∗∗^*p* < 0.0001.

A significant main effect of RIS treatment on NO [*F*_(3,20)_ = 15.28, *p* < 0.001] level was observed in the prefrontal cortex (**Figure 3A**). *Post hoc* analysis showed an enhanced level of NO in the LPS (*M* = 7.72, *SD* = 3.07) than the control (*M* = 1.84, *SD* = 0.19) group. LPS+RIS (*M* = 4.23, *SD* = 0.88) group showed a decreased level of NO than LPS (*M* = 7.72, *SD* = 3.07) group.

There was a noticeable main effect of treatment on NO [*F*_(3,20)_ = 259.2, *p* < 0.001] level in the hippocampus (**Figure 3B**). *Post hoc* test disclosed an increased level of NO in the LPS group (*M* = 7.60, *SD* = 0.61) as compared to control (*M* = 1.83, *SD* = 0.38). LPS+ RIS (*M* = 3.02, *SD* = 0.34) group mice showed a diminished level of NO than LPS (*M* = 7.60, *SD* = 0.61) only group. As shown in **Figure 3C**, a significant main effect of treatment on NO [*F*_(3,19)_ = 32.74, *p* < 0.001] was observed in the liver also. *Post hoc* study showed a higher level of NO in the LPS (*M* = 6.80, *SD* = 0.32) than the control (*M* = 3.55, *SD* = 1.19) group. However, no significant difference was found between LPS+RIS (*M* = 6.48, *SD* = 0.23) and LPS mice groups.

### Effect of Risperidone on Catalase (CAT) Level

**FIGURE 4:**
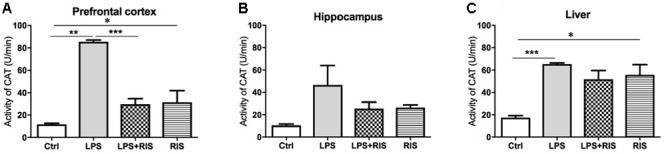
Effect of various treatments on the activity of catalase (CAT). Groups are Ctrl (control), LPS (lipopolysaccharide), LPS+RIS (lipopolysaccharide + risperidone), and RIS (risperidone). CAT activity was measured from the prefrontal cortex **(A)**, hippocampus **(B)**, and liver **(C)** tissues. Values are represented as mean ± SEM. *Post hoc* multiple comparison test namely, “Newman–Keuls” test was used to compare between groups. *n* = 6 per group. ^∗^*p* < 0.05, ^∗∗^*p* < 0.01, ^∗∗∗^*p* < 0.001.

The main effect of treatment on CAT was noteworthy [*F*_(3,17)_ = 53.59, *p* < 0.001] in the prefrontal cortex (**Figure 4A**). *Post hoc* analysis indicated an increased level of CAT in the LPS (*M* = 85.50, *SD* = 3.83) than the control (*M* = 11.69, *SD* = 2.36) group. Importantly, LPS+ RIS (*M* = 29.73, *SD* = 11.16) mice had a reduced level of CAT than LPS (*M* = 85.50, *SD* = 3.83) only group.

There was no significant main effect of treatment on CAT [*F*_(3,20)_ = 2.58, *p* = 0.08] in the hippocampus (**Figure 4B**). However, there was an obvious main effect of treatment on CAT [*F*_(3,19)_ = 9.68, *p* < 0.001] in the liver (**Figure 4C**). *Post hoc* multiple comparison analysis exhibited a higher activity of CAT in the LPS (*M* = 65.34, *SD* = 2.55) than control (*M* = 17.48, *SD* = 3.85), LPS+RIS (*M* = 51.78, *SD* = 19.26) and RIS (*M* = 55.67, *SD* = 22.59) groups.

### Effect of Risperidone on Superoxide Dismutase (SOD) Level

**FIGURE 5:**
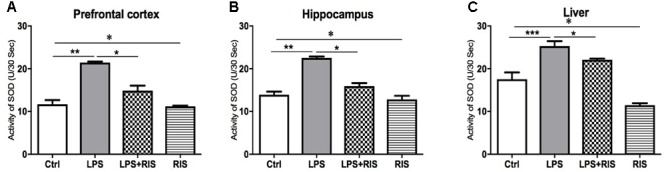
Effect of various treatments on the activity of superoxide dismutase (SOD). Groups are Ctrl (control), LPS (lipopolysaccharide), LPS+RIS (lipopolysaccharide + risperidone), and RIS (risperidone). SOD activity was measured from the prefrontal cortex **(A)**, hippocampus **(B)**, and liver **(C)** tissues. Values are represented as mean ± SEM. *Post hoc* multiple comparison test namely, “Newman–Keuls” test was used to compare between groups. *n* = 6 per group. ^∗^*p* < 0.05, ^∗∗^*p* < 0.01, ^∗∗∗^*p* < 0.001.

Risperidone treatment showed a significantly strong main effect on SOD level [*F*_(3,20)_ = 36.32, *p* < 0.001] in the prefrontal cortex (**Figure 5A**). *Post hoc* analysis exhibited an increased SOD level in the LPS (*M* = 21.43, *SD* = 0.61) than the control (*M* = 11.69, *SD* = 2.37) group. LPS+RIS (*M* = 14.86, *SD* = 2.91) mice showed a significantly lower level of SOD than LPS (*M* = 21.43, *SD* = 0.61) which demonstrates the effectiveness of the treatment.

The main effect of treatment on SOD [*F*_(3,20)_ = 40.94, *p* < 0.001] level in the hippocampus is shown in **Figure 5B**. *Post hoc* analysis exhibited a significantly higher SOD level in the LPS (*M* = 22.54, *SD* = 0.78) than control (*M* = 13.92, *SD* = 1.75). Interestingly, LPS+RIS (*M* = 15.94, *SD* = 0.24) mice showed a lower SOD activity than LPS (*M* = 22.54, *SD* = 0.78) mice. There was a noticeable main effect of treatment on SOD [*F*_(3,20)_ = 34.81, *p* < 0.001] in the liver (**Figure 5C**). *Post hoc* test revealed a significant elevation of SOD in LPS group (*M* = 25.30, *SD* = 2.79) than the control (*M* = 17.54, *SD* = 3.91). By contrasts, LPS+RIS (*M* = 22.08, *SD* = 0.70) group showed a reduced SOD level than LPS group.

### Effect of Risperidone on the Level of Glutathione (GSH)

**FIGURE 6:**
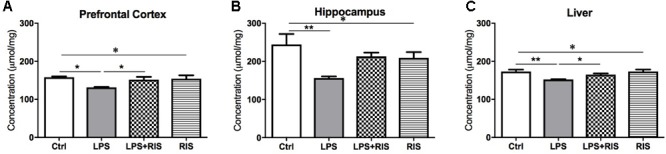
Effect of various treatments on the level of glutathione (GSH). Groups are Ctrl (control), LPS (lipopolysaccharide), LPS+RIS (lipopolysaccharide + risperidone), and RIS (risperidone). GSH was measured from the prefrontal cortex **(A)**, hippocampus **(B)**, and liver **(C)** tissues. Values are represented as mean ± SEM. *Post hoc* multiple comparison test namely, “Newman–Keuls” test was used to compare between groups. *n* = 6 per group. ^∗^*p* < 0.05, ^∗∗^*p* < 0.01, ^∗∗∗^*p* < 0.001.

There was a significant main effect of treatment on GSH [*F*_(3,20)_ = 4.19, *p* = 0.018] level in the prefrontal cortex (**Figure 6A**). *Post hoc* multiple comparison analysis displayed a reduced level of GSH in the LPS (*M* = 131.5, *SD* = 2.74) group than the control (*M* = 158.2, *SD* = 5.49). By contrasts, LPS+RIS (*M* = 151.8, *SD* = 17.72) mice showed a higher glutathione level than LPS mice.

There was a noticeable main effect of treatment on GSH [*F*_(3,20)_ = 4.75, *p* = 0.011] level in the hippocampus (**Figure 6B**). *Post hoc* test revealed a significantly reduced level of GSH in the LPS (*M* = 156.3, *SD* = 10.60) than the control (*M* = 244.3, *SD* = 67.65) group. Moreover, LPS+RIS (*M* = 213.0, *SD* = 24.55) mice showed no significant difference than LPS treated mice.

There was a significant main effect of treatment on GSH [*F*_(3,20)_ = 5.86, *p* = 0.004] level in the liver (**Figure 6C**). *Post hoc* test displayed a significant reduction of GSH in LPS (*M* = 152.3, *SD* = 1.37) than control (*M* = 173.0, *SD* = 12.68). In addition, LPS+RIS (*M* = 165.0, *SD* = 7.64) mice showed a significantly higher level of GSH than LPS mice.

## Discussion

The present study was designed to evaluate the neuroprotective effect of risperidone pre-treatment on the acute peripheral LPS-induced oxidative damage in different regions of the brain in *Swiss albino* mice. We found that the level of oxidative damage in the prefrontal cortex and hippocampus were markedly enhanced following intraperitoneal LPS administration. However, pretreatment with risperidone improved most of the oxidative stress parameters in the hippocampus and cortex.

Our results showed that LPS causes a significant detrimental effect on the prefrontal cortex and hippocampus by increasing the breakdown of lipids and proteins. Moreover, LPS enhanced the level of NO and diminished the level of glutathione. In response to this damage, the level of antioxidant enzymes, catalase and SOD were elevated It is possible that the administration of LPS increased the activity of antioxidant enzymes in the brain regions. Our results are consistent with the previous findings (Al-Amin et al., 2013; MacDowell et al., 2013; Cauwels et al., 2014; Jia et al., 2014; Su et al., 2014; Zhang et al., 2015). We also found that RIS treatment reduces LPS-induced increased MDA level in the hippocampus but not in the liver. Also, LPS+RIS group showed reduction in LPS-induced increase in NO levels in the hippocampus and cortex. These results are consistent with the studies performed on RIS (Shioda et al., 2012). Furthermore, LPS+RIS improved the level of AOPP in the cortex and hippocampus. Our results indicate that RIS treatment increased the level of glutathione in the cortex and liver, which is in agreement with a previous study (Stojković et al., 2012).

The effect of RIS treatment on the level of glutathione in LPS-induced neuroinflammatory model has not been studied in the past. However, a previous study reported that LPS reduces the level of glutathione (Yamada et al., 2006). In addition, RIS could reverse phencyclidine-induced reduced glutathione levels in the rat brain (Stojković et al., 2012). Consistent with this finding, we also report that RIS may reverse LPS-induced reduced glutathione levels.

Upon analyzing the levels of the antioxidant enzymes catalase and SOD, we found that RIS pretreatment improves the activity of catalase in the cortex and hippocampus.

Our observation that the level of SOD was improved in the brain is consistent with several previous studies (Zhang et al., 2012; Yan et al., 2014). All these findings taken together, asserts that pretreatment with RIS can rescue the LPS-induced oxidative stress in the brain.

The damaging effect of LPS due to oxidative stress in the brain has been demonstrated in the previous studies as well (Shukla et al., 2008; Del Angel-Meza et al., 2011; Tomaz et al., 2014; Ho et al., 2015). The organelle that encounters the oxidative stress most is the mitochondria. Mitochondria produce energy by the breakdown of glucose with the help of oxygen. It is noted that the brain requires almost 20% of the total oxygen intake to produce energy. Mitochondria in the neural cells are constantly producing highly reactive free radicals as by product during the metabolic processes. The excess amount of free radicals may cause the damage to proteins, lipids and even the neural function (Lohr, 1991; Lohr and Browning, 1995). Unfortunately, neural cells are poorly equipped to defend against oxidative damage (Mahadik and Mukherjee, 1996). The underlying reason of this poor defensive system is due to the sensitivity of lipids to the free radicals, failure of adult neurons to reproduce and compensate destroyed DNA and poor activity of antioxidant enzymes namely glutathione, catalase, and SOD (Cohen, 1984). Impaired antioxidant defense system has been reported to be associated with neuropsychiatric diseases (Lohr, 1991; Lohr and Browning, 1995; Salim, 2014), such as, schizophrenia (Li et al., 2006), autism (McGinnis, 2004), depression (Michel et al., 2012), bipolar mood disorder (Erdem et al., 2014). In this study, treatment with risperidone showed decrease in the LPS-induced high levels of oxidative markers in the distinct brain regions. We report that the administration of RIS in LPS-induced stress model might be promising in improving the deficits due to oxidative stress. Accumulating evidences suggest the neuroprotective potential of RIS (Lauterbach et al., 2010) via antioxidant immunomodulatory activity. A study with 22 schizophrenic patients demonstrated that RIS possesses significant antioxidant activity (Gilca et al., 2014). Moreover, RIS treatment lowers lipid peroxidation on 51 first episode schizophrenic patients treated with risperidone for 11 weeks. Noto et al. (2015), suggested that RIS enhances antioxidant defense against lipid peroxidation possibly through PON1 (paraoxonase 1) antioxidant enzyme. Immunomodulatory potential of RIS was observed when given with celecoxib as an add-on therapy in schizophrenia (Müller et al., 2002). Animal model of gerbil stroke revealed that post-treatment with RIS provides neuroprotection presumably via attenuation of microglial activation as well as maintenance of the antioxidants (Yan et al., 2014). RIS also shows the antioxidant activity by lowering the glutathione level in phencyclidine-treated rat brain (Stojković et al., 2012). RIS has also been found effective in preventing microglia activation and improves behavioral deficits when LPS was administered directly to the hippocampus in neonatal rats (Zhu et al., 2014b).

In this study, LPS was administered only once on day 8. We have observed that acute LPS administration increased immobility time in the behavioral test. We have not incorporated behavioral data in this study since acute LPS administration causes immediate effect on behavioral level. We have conducted open field test and tail suspension test; both tests have shown a significant increase in immobile phase following LPS administration (data not shown) as consistent with the previous studies (Frenois et al., 2007; O’Connor et al., 2008). LPS at a dose of 25, 50, 100, or 1000 μg/kg caused suppressed behavior in the open field test (Yirmiya et al., 1994), while LPS at 0.63 or 1.25 μg/kg exhibited diminished locomotor activity at 2 h (Dunn and Swiergiel, 2005), reduced distance traveled at 6 h and poor exploration behavior in 24 h (Biesmans et al., 2013). It is noted that we used 0.8 mg/kg (800 μg/kg) (El-Sayed and Bayan, 2015) single dose to induce behavioral impairment. LPS at a dose of 1 and 5 μg/kg once dose has been shown to produce long immobility time in the TST (Dunn and Swiergiel, 2005).

Lipopolysaccharide directly enhances the production of inflammatory cytokines. Therefore, the determination of the cytokines in this study could have provided insights into the effect of RIS in the LPS-induced impairment at the molecular level. Despite this limitation, our study is novel in some respects. To the best of our knowledge, this study is the first to report the role of RIS in the LPS-induced six oxidative stress markers in the two important brain regions that are involved in cognitive function, learning and memory formation. Moreover, liver data provided additional evidence on overall condition besides the brain. Our findings further support the hypothesis that RIS possesses a significant antioxidant property that could be studied further in details. There are several possible explanations in favor of the current outcomes (i) RIS may regulate the production of GSH, CAT, and SOD to combat against ROS, (ii) RIS might diminish the formation of ROS, (iii) RIS, itself is involved in neutralizing the already formed ROS, (iv) RIS is possibly associated with the regulation of GSH, SOD, and CAT forming genes. Future work can be undertaken to gain insight into the mechanisms underlying the antioxidant activity of RIS in improving the stress condition induced by LPS.

## Conclusion

The present study was designed to determine the effect of risperidone on systemic LPS-induced oxidative damage in the specific brain regions and liver. The study has demonstrated that risperidone is effective in reversing LPS-mediated behavioral impairment and subsequent oxidative stress. In conclusion, our findings suggest that risperidone may possess some neuro-protective effects through reduction of oxidative stress parameters. The results of this research support the notion that RIS diminishes the production of ROS possibly through the regulation of antioxidant enzymes in the brain. We further propose that RIS may contribute to the improvement in brain diseases due to its protective effects against the endotoxin-linked oxidative damage in the brain.

## Author Contributions

MAA-A and HMR participated in the design of the experimental work. MAA-A, MFRC performed experiments and analyzed the data. ASC and TRC provided technical support. PJ and HMR contributed to preparation, editing and critically revising the manuscript for important intellectual content. MK, MA and HMR corrected drafts and obtained the funding. SMA contributed in the manuscript during the revision period and shared his experience in editing the manuscript. All authors read and approved the final manuscript.

## Conflict of Interest Statement

The authors declare that the research was conducted in the absence of any commercial or financial relationships that could be construed as a potential conflict of interest.
